# Effect of Genetic Information and Information About Caffeine Content on Caffeine Withdrawal Symptoms

**DOI:** 10.1038/s41598-017-08678-4

**Published:** 2017-08-21

**Authors:** Llewellyn Mills, Ilan Dar-Nimrod, Ben Colagiuri

**Affiliations:** School of Psychology, University of Sydney, Sydney, Australia

## Abstract

This study sought to test the effect of genetic information and information about the caffeine content of a beverage on caffeine withdrawal, specifically if: (1) being informed that one has tested positive for a gene related to caffeine withdrawal can produce an exaggerated caffeine withdrawal response during abstinence; (2) belief that one has consumed caffeine leads to a reduction in withdrawal symptoms when no caffeine is consumed. Regular coffee drinkers were given a bogus genetic test and were told either that they had tested positive or negative for a gene related to withdrawal. After 24-hour caffeine abstinence withdrawal symptoms were measured using a self-report caffeine withdrawal scale, and then again after a cup of decaffeinated coffee. Half the participants were told their coffee was caffeinated and half were told truthfully that it was decaffeinated. Participants told the coffee was caffeinated reported a greater reduction in withdrawal symptoms than those told it was decaffeinated. Differing genetic test result information produced no difference in reported withdrawal symptoms. These results indicate that information about the dose of caffeine administered can influence withdrawal symptoms, but that genetic information does not have a universal ability to produce nocebo effects across all sensory and cognitive domains.

## Introduction

Finding out we have a gene associated with a medical condition can exert a powerful influence on our thoughts, emotions, and behaviour. According to the genetic-essentialism framework^1^, genetic explanations of health outcomes can induce attributional biases that alter perceptions of those outcomes, so that they are seen as more enduring, unchangeable, and innate. These biases mean that when individuals are told they possess a gene known to be associated with a condition it changes the way they assess the risk of developing that condition in the future. The change in perceived risk prompts an increase in disease-specific negative affect, such as worry about the future risk and consequences of developing the condition^2–5^, which in turn increases voluntary behaviours that might reduce the risk of developing the condition in future, such as screening for the relevant condition or enrolling in educational workshops^6–10^ or changing insurance arrangements to minimize the impact of the adverse condition should it occur^11^. These voluntary behavioural changes are consistent with an increase in regularity of thought concerning the relevant condition brought about by discovering one has the gene.

Most studies examining the consequences of receiving ‘gene-positive’ information have focused on volitional outcomes such as those mentioned above. However the increase in thought and worry about the condition may also affect non-volitional outcomes via the nocebo effect. The nocebo effect is the occurrence of negative consequences following a treatment that are attributable to factors other than the innate properties of the treatment itself. For example self-reported pain can be increased by simply suggesting that an agent will cause an increase in pain sensitivity^12^ or even just that it will heighten awareness of bodily sensations^13^.

To date, two studies have reported what could be interpreted as nocebo effects following the delivery of gene-positive information. One study by Dar-Nimrod and Colleagues found that healthy participants who were informed they possessed a gene associated with alcoholism following a bogus genetic test reported a significantly greater increase in negative affect and significantly lower reduction in positive affect than those told they did not possess the gene^8^. Whether this result could be termed a nocebo effect depends on the nature of the negative affect. A *general* increase in negative affect brought about by information alone is consistent with the definition of a nocebo effect but negative affect confined solely to worry about future risk of alcoholism is not. Because the scale used to measure negative and positive affect in that study was of a general nature, it is difficult to determine whether their result indicates a nocebo effect or an increase in disease-specific distress. A second study, by Lineweaver and Colleagues, found that participants who genuinely tested positive for a gene related to Alzheimer’s disease and were informed of the result scored lower on a memory test and judged their own memory more harshly than participants who also tested positive for the gene but were not informed^14^. As memory impairment is widely known as the hallmark symptom of Alzheimer’s disease, this result suggests that receiving the gene-positive information served as a type of ‘memory-loss prime’ that elicited an objective, non-volitional response that was congruent with the prime.

Priming involves exposure to verbal or contextual cues that activate a mindset which subsequently influences the attention to and interpretation of stimuli that are relevant to the primed construct^15^, and is one potential mechanism of the nocebo effect. Primes are thought to activate expectancies that bias attention towards the somatosensory domains that are relevant to those expectancies, leading to an amplification of otherwise normal levels of symptoms in these domains, and a resultant nocebo effect^16–19^. Thus Lineweaver and Colleagues’ finding could be interpreted as gene-positive information inducing or ‘priming’ a nocebo effect. If viewed this way their finding is consistent with other studies, in areas unrelated to genetic testing, where priming manipulations have brought about nocebo effects. For example being exposed to warnings about side-effects of a medication on a consent form can increase reporting of cognitive deficits in chemotherapy patients^17^, gastrointestinal complaints in treatment of unstable angina with aspirin^20^, and insomnia in participants reporting sleep difficulty^21^.

Given the dramatic rise in the availability of genetic testing for disease risk, and the possibility that this information could create nocebo effects, it is important to determine what conditions are sensitive to genetic information primes. Drug withdrawal is a condition that is particularly suited to test the priming effect of genetic information, for several reasons. First addiction is a behavioural tendency that has been linked to genes and heritability^22–24^ which means that addiction-related phenomena such as withdrawal can be plausibly presented as heritable and linked to a specific gene. Secondly there is evidence that the experience of withdrawal symptoms can be influenced by expectancies about having ingested a drug or not^25–28^. For example we found that abstinent coffee drinkers who were given a cup of decaffeinated coffee reported a greater reduction in withdrawal symptoms if they were told that the coffee was caffeinated than if they were told truthfully it was decaffeinated^29^.

This current study therefore aimed to (1) determine whether information indicating genetic susceptibility to caffeine withdrawal symptoms produces a heightened caffeine withdrawal response, and (2) to test whether this withdrawal-susceptibility information interacts with the ability of placebo caffeine to reduce withdrawal symptoms. Caffeine withdrawal is an ideal form of withdrawal upon which to test the priming potential of genetic information because: (1) it is an addictive drug with an established and withdrawal syndrome^30^, (2) it consumed by 80–90% of the adult population^31^, (3) its withdrawal syndrome is relatively mild and thus experimental expectancy manipulations can be conducted ethically with less personal repercussions for participants. To our knowledge this study is the first to use genetic information to prime a nocebo effect in a healthy population.

## Methods

### Design

**Figure 1:**
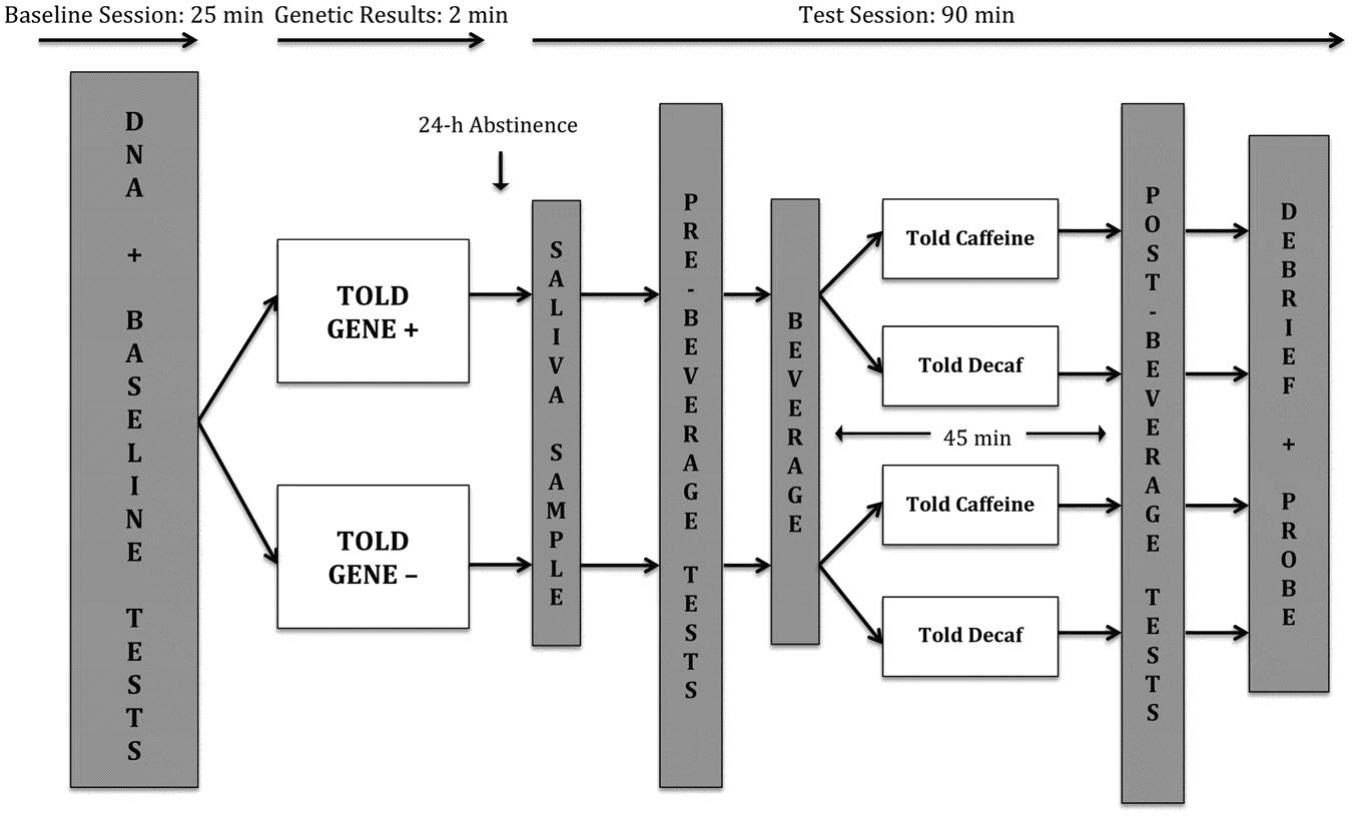
Study Design. The Gene + group were told they had tested positive for a gene associated with heightened caffeine withdrawal symptoms. The Gene− group were told they had tested negative for this gene. The Told Caffeine group were told they were receiving caffeinated coffee. The Told Decaf group was told truthfully that they were receiving decaffeinated coffee. Baseline, Pre-Beverage and Post-Beverage tests were, in order: 1) Blood Pressure; 2) Score on the Caffeine Withdrawal Symptom Questionnaire (CWSQ; Juliano *et al*., 2012); 3) Score on the RVIP task, a test of concentration.

The study used a 2 × 2 × (2) mixed design, as shown in Fig. 1. There were two two-level, between-subjects factors: Priming (Gene + vs Gene−conditions) and Caffeine Information (Told Caffeine vs Told Decaf condition). The two-level within-subjects factor was Time, with one measurement being taken after 24 hours abstinence from caffeine (Pre-Beverage) and the other being taken 45 minutes after a cup of decaffeinated coffee (Post-Beverage). The primary outcome of interest was self-reported caffeine withdrawal symptoms. Secondary outcome variables blood-pressure readings and a test of concentration were also recorded at both time points however these variables were included primarily to heighten the impression of the ‘medical/psychophysiological’ nature of the study and were not the study’s main focus.

### Participants

Ninety-three regular coffee drinkers (38 male; average age = 29.9; range = 18–64) were paid $50 in exchange for their participation. Participants were included in the study if they consumed at least 270 mg (approximately three 180 ml cups of roasted or ground coffee^32^) per day, were 18 years of age or over, and had sufficient written and verbal English skills. Participants gave informed consent to take part in a study investigating the effect of genes on mood during caffeine abstinence, but were fully debriefed as to the study’s true purpose upon completion.

### Materials and Measures

#### Demographic and Caffeine Use Questionnaire

Demographic information and daily caffeine consumption across all beverages was ascertained via a computerised questionnaire. Estimates of caffeine content of beverages were obtained from Barone and Roberts’^32^ study listing caffeine content of popular beverages, or from content listed by the manufacturer on their websites or directly on packaging.

#### Caffeine Withdrawal Symptom Questionnaire

A computerised version of the Caffeine Withdrawal Symptom Questionnaire (CWSQ^33^) was used to assess withdrawal symptoms. The 32-item CWSQ is arranged in nine separate factors: Drowsiness/Fatigue; Decreased Alertness/Difficulty Concentrating; Mood Disturbances; Decreased Sociability/Motivation to Work; Nausea/Upset Stomach; Flu-like Feelings; Headache, Acute Caffeine Effects, and Craving. Participants were asked to rate to what extent they were experiencing each symptom on a 5-point response scale from 0 (‘not at all’) to 4 (‘extremely’). The maximum possible score was 128.

#### Rapid Visual Information Processing task (RVIP)

Participants were given a version of the RVIP, a test of sustained attention that has proven sensitive to placebo caffeine manipulations^34^. In this 5-minute task participants were required to monitor single digits appearing on a screen in semi-random order and to detect strings of three consecutive odd or three consecutive even digits amongst the random digits. Performance on the test was measured via hit rate, false-alarm rate, reaction time, and a composite accuracy score.

#### Blood Pressure

Systolic and diastolic blood pressure was measured via an Omron HEM-7221 electronic sphygmomanometer.

#### Exit Questionnaire/Manipulation Check

A computerised exit questionnaire was used; (1) to probe participants for suspicions about the veracity of the genetic test results or the stated caffeine content of their beverage (2) to obtain biohistorical information such as years of daily caffeine use and number of previous attempts to quit or reduce caffeine intake.

#### Bogus DNA-Test

Each participant provided a saliva sample via Isohelix SK-2S buccal mouth swabs. Swabs were stored in a GVA 42-L bar refrigerator housed in the test room.

#### Beverages

Coffee was prepared in a DeLonghi Magnifica Automatic Coffee Machine using Peet’s Major Dickason’s Blend decaffeinated coffee beans. These beans contain approximately 4% of the caffeine content of regular caffeinated coffee beans, amounting to 4 mg or less of caffeine per cup. Coffee was served in 227-ml Keji double wall paper coffee cups, each cup containing approximately 180 ml of liquid.

### Procedure

#### Recruitment

Participants were recruited via an online classifieds website or on the University of Sydney Careers website. Advertisements requested participants for a study testing the effect of genes on mood during caffeine abstinence. As a way of ensuring participants were honest about their caffeine use no mention was made in the ad of the minimum daily consumption required for inclusion in the study. Advertisements asked interested participants to email the researchers telling them how many cups of coffee they drank on an average weekday. If participants met the minimum daily coffee consumption criteria (equivalent of three 180 ml cups of roasted or ground coffee) they were sent a return email saying they qualified for the study and detailing the general study procedures.

#### Ad-Libitum Baseline Session

Prior to attendance participants were instructed that on the day of the Baseline session they should consume caffeine as they normally would. When a participant first arrived a researcher verbally told them the cover story, which was: (1) that studies had found a link between presence of the A1 allele of the Dopamine D2 receptor gene (DRD2) and addiction to various drugs (2) that this was thought to occur because people who possess this gene experience more severe withdrawal symptoms during abstinence than people who do not possess the gene (3) that this had been found to be true for several addictive drugs, including caffeine. Participants were then informed of the most common caffeine withdrawal symptoms. The specific gene used in the cover was chosen because it has been shown to be a possible risk factor in the development of addiction, and hence would withstand scrutiny if participants chose to research it independently. In addition participants were asked to read an information sheet containing a written version of the cover story that they took with them at the completion of the session. Participants then completed the Demographic and Caffeine Use questionnaire. Following this participants completed the primary test battery: CWSQ, RVIP, and blood pressure. When baseline tests were completed participants provided a tissue sample via buccal mouth swab, which they were told would be analysed and the results delivered directly to them by phone in a few days. Swabs were not analysed in any way.

#### Delivery of ‘Results’ of Genetic Test

Prior to delivery of the bogus test results participants were randomly assigned to either the Gene + or Gene−groups. Results were predominately delivered 24–36 hours prior to participants’ test session, however occasionally participants had to reschedule this session in which case the duration between receiving results and testing was longer. To account for these differences, duration between ‘results’ and test was recorded to be used as a covariate in analyses. Researchers delivered the bogus results by reading a pre-written script directly to participants over the phone. In this script participants were told either that they had tested positive or negative for presence of the A1 allele. The link between the allele and addiction/withdrawal was then re-iterated. Participants informed that they were gene-positive were told that they may be at a heightened risk for suffering caffeine withdrawal symptoms, whereas participants informed they were gene-negative were told they were unlikely to have a genetically heightened risk for experiencing caffeine withdrawal symptoms. Once again these caffeine withdrawal symptoms were read out to participants. Participants were then asked if they understood the results and if they had any questions. If participants had questions these were answered as briefly as possible and in line with the cover story. Participants were then reminded of their test session appointment and that they were required to remain abstinent from all sources of caffeine (these sources were listed) for 24-h prior to the test session. Using a ‘bogus pipeline’ procedure to enhance compliance with this requirement^35^ participants were told that caffeine abstinence would be verified upon arrival via a saliva test.

#### Test Session

Participants were tested individually in a single 90-min test session. Prior to this session they were randomly allocated to either the Told Caffeine or Told Decaf conditions. Upon arrival participants had saliva samples collected (these samples were not analysed). Following saliva collection participants were administered the primary test battery (RVIP, CWSQ, and blood-pressure). Next, participants were given their cup of coffee, which was prepared in front of them in the test room. All participants received decaffeinated coffee. Beans used to make participants’ coffee were placed in the test room prior to participants’ arrival according to group allocation; either in original packaging or in decoy packaging of an inexpensive popular (caffeinated) blend. During preparation of the coffee, participants in the Told Decaf group were instructed that they had been allocated to a control condition of the study and would therefore receive decaffeinated coffee. The Told Caffeine group on the other hand were not given any further instructions, since all participants had been led to believe that they would be receiving caffeinated coffee as part of the general study description. Participants then consumed their coffee, after which they were allowed a 45-minute ‘caffeine absorption period’ in which they remained in the lab but were free to study, browse the internet, or use their smartphones. Following the ‘absorption period’ participants had their blood pressure read and took the RVIP and CWSQ tests a second time. Finally, participants were given the exit questionnaire and debriefed as to the true nature of the study. All the procedures in this study were approved by the University of Sydney Human Research Ethics committee and were conducted in accordance with the 1964 declaration of Helsinki.

### Data Availability

Data will be made available to interested readers upon request with proper authorization from mutual ethics committees.

## Results

### Exclusions

Thirteen participants were excluded from analyses. Participants were excluded: (1) if they gave any indication, either in the post-test in-person debrief or in the computer-based exit questionnaire, that they had suspicions about the genetic test prior to or during testing, or (2) if they were suspicious about the instruction given to them about the caffeine content of their beverage for any reasons other than their withdrawal symptoms (e.g. a general distrust of psychology experiments). This left 80 participants upon whose data analyses were performed (*n* = 20 participants in each of the four groups). Six of these participants indicated in the exit questionnaire that they believed the caffeine content of their beverage was different what they were told (Told Caffeine group = 4/40; Told Decaf group = 2/40); however these participants were not excluded from analysis because, when probed further during the debrief session, they indicated reasons for their belief that related to the way they felt after their coffee (e.g. ‘because I did not feel any kick’ or ‘because I felt more awake after drinking the coffee’) rather than any general suspicions relating to the specific experiment or to psychological research overall. As there was no significant difference in this proportion between the Told Caffeine and Told Decaf group (χ^2^_(1)_ = 0.18, *p* = 0.671) these participants were analysed in the group they were allocated to.

### Participant Characteristics

Mean daily caffeine consumption was 296.9 mg (SD = 120.5) per day. All participants indicated that their primary source of caffeine was from coffee, with an average consumption of 3.1 180- ml cups of roasted or ground coffee per day (SD = 1.2). The modal number of attempts at quitting caffeine prior to enrolling in the study was 1 (M = 2.0, SD = 3.6). The mean number of years of daily caffeine use was 11.9 (SD = 10.8).

### Primary Outcome: Caffeine Withdrawal Symptoms

A 2 × 2 × (2) mixed-model ANCOVA was performed, with Priming (Gene−vs Gene + ), Caffeine Information (Told Caffeine vs Told Decaf), and Time (Pre-Beverage vs Post-Beverage) as the independent variables. From a review of caffeine and addiction literature several biohistorical factors were identified that could potentially create differences in withdrawal levels across groups and individuals. These factors were (1) total amount of caffeine consumed each day, (2) number of past attempts to quit or cut back caffeine consumption, (3) number of years of regular (i.e. daily) caffeine use. These three factors, along with Baseline CWSQ score (4) and duration in days between delivery of ‘results’ and test (5), were entered as covariates in the mixed ANCOVA. The primary outcome variable was total score on the CWSQ, however analysis of RVIP scores and blood-pressure were also conducted. Assumptions tests performed on both Pre- and Post-Beverage CWSQ scores indicated normality (Shapiro-Wilks test; Pre: *W* = 0.99, *p* = 0.82; Post: *W* = 0.97, *p* = 0.09) and homogeneity of variance (Kruskal-Wallis test on four experimental groups: Pre: *χ* ^2^_(3)_ = 5.33, *p* = 0.15; Post: *χ* ^2^_(3)_ = 4.6, *p* = 0.20) were upheld.

### Effect of Priming and Caffeine Information on Withdrawal Symptoms

**Figure 2:**
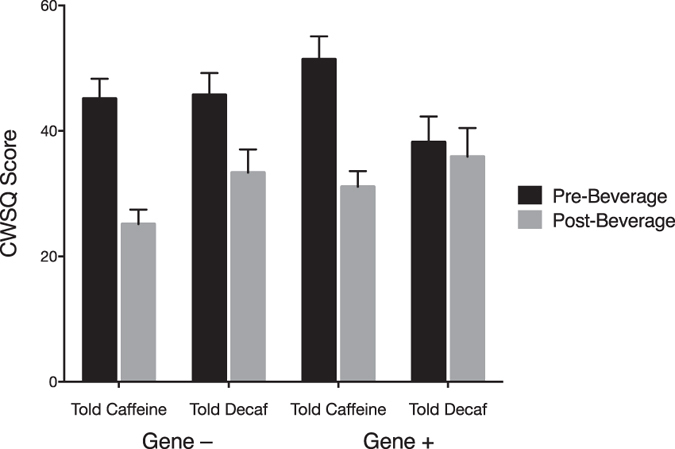
Effect of Time, Priming, and Caffeine Information on Caffeine Withdrawal Symptoms. Error bars indicate standard error.

**Figure 3:**
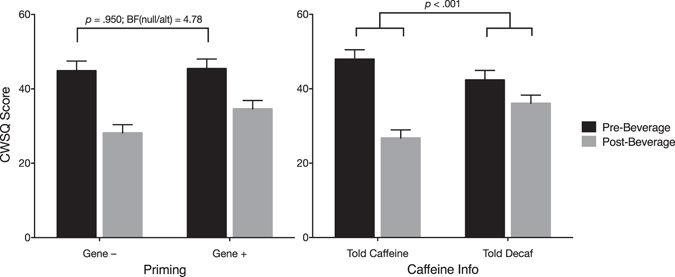
Effect of interaction between Time and Priming (left) and Time and Caffeine Information (right) on Caffeine Withdrawal. Error bars indicate standard error.

Mean CWSQ scores across time points and groups are presented in Table 1. Figure 2 shows mean Pre- and Post-Beverage CWSQ scores across groups and Fig. 3 shows the main effects of Priming and Caffeine Information.

**Table 1:** Descriptive Statistics for Caffeine Withdrawal Across Time Points and Groups.

	mean	sd	n
Time			
Pre-Beverage	45.13	±16.6	80
Post-Beverage	31.35	±15.4	80
Priming			
Gene +	39.15	±18.2	40
Gene −	37.35	±16.5	40
Caffeine Information			
Told Caffeine	38.2	±16.8	40
Told Decaf	38.3	±18.1	40
Priming × Time			
Gene +/Pre	44.8	±18.4	40
Gene −/Pre	45.45	±14.7	40
Gene +/Post	33.5	±16.4	40
Gene −/Post	29.25	±14.2	40
Caffeine Information × Time			
Told Caffeine/Pre	48.28	±15.5	40
Told Decaf/Pre	41.98	±17.2	40
Told Caffeine/Post	28.13	±11.0	40
Told Decaf/Post	34.63	±18.4	40
Priming x Caffeine Information × Time			
Gene +/Told Caffeine/Pre	51.4	±16.4	20
Gene + /Told Decaf/Pre	38.2	±18.4	20
Gene −/Told Caffeine/Pre	45.15	±14.2	20
Gene −/Told Decaf/Pre	45.75	±15.6	20
Gene +/Told Caffeine/Post	31.1	±11.2	20
Gene + /Told Decaf/Post	35.9	±20.4	20
Gene −/Told Caffeine/Post	25.15	±10.3	20
Gene −/Told Decaf/Post	33.35	±16.6	20

### Effect of Time

The effect of Time on caffeine withdrawal symptoms was significant (${F}_{(\mathrm{1,71})}$ = 6.241; *p* = 0.015, ${\eta }_{p}^{2}$ = 0.081) with participants experiencing a 13.75-point reduction in withdrawal symptoms on average from Pre- to Post-Beverage.

### Effect of Priming

As it was hypothesised that the two different test results (Gene + vs Gene−) would induce a difference in perceived caffeine withdrawal after 24-h abstinence, the key test was differences in *Pre-Beverage* CWSQ scores according to type of genetic information. Mean Pre-Beverage CWSQ score was 44.8 (SD = 18.42) in the Gene + group and 45.45 (SD = 14.74) in the Gene – group (see black bars in Figure 3-left), a non-significant difference of 0.65 points (${F}_{(\mathrm{1,71})}$ = 0.004; *p* = 0.950, ${\eta }_{p}^{2}$ < 0.001). Given the extremely low *F* and ${\eta }_{p}^{2}$ statistics, a post-hoc Bayes Factor test was performed. Bayes factors are an alternative form of hypothesis testing that allow direct comparisons of null hypotheses to alternative hypotheses, thus allowing researchers to quantify evidence for the *absence* of an effect (an inference that cannot be made with tradition null hypothesis significance testing). Bayes Factors >3.2 in favour of one hypothesis over another constitute ‘substantial’ evidence for that hypothesis^36^. We used the *BayesFactor* package^37^ in R^38^ to test the null hypothesis of no difference in Pre-Beverage CWSQ scores between the Gene+ and Gene – groups against the directional alternative hypothesis of the Gene + group having a higher withdrawal level at the Pre-Beverage test. The prior distribution for the alternative hypothesis was a folded Cauchy distribution with scale parameter = √2/2 (see *BayesFactor* package manual)^37^ which places more prior credibility on smaller effect sizes. This yielded a Bayes Factor of 4.87. Thus it is 4.87 times more likely, given the data, that there was no difference in Pre-Beverage CWSQ scores due to the Priming manipulation (null hypothesis) than that the Gene + group had a slightly higher level of withdrawal (alternative hypothesis).

The interaction between Time and Priming was not significant (${F}_{(\mathrm{1,71})}$ = 3.21; *p* = 0.078, ${\eta }_{p}^{2}$ = 0.043).

### Effect of Information About Caffeine Content of Beverage

The interaction between Caffeine Information and Time was significant (${F}_{(\mathrm{1,71})}$ = 22.86; *p* < 0.001, ${\eta }_{p}^{2}$ = 0.244; see Fig. 3-right) with participants in the Told Caffeine group experiencing a reduction in CWSQ score from Pre- to Post-Beverage that was 14.6 points greater on average than the Told Decaf group.

There were no significant three-way interactions for CWSQ scores.

### Post-Hoc Tests

There appeared to be numerical differences in Pre-Beverage CWSQ scores across the four groups, particularly between the Gene +/Told Caffeine (Pre-Beverage mean = 51.4; SD = 16.37) and the Gene +/Told Decaf group (Pre-Beverage mean = 38.2; SD = 18.35). The only difference in these groups is Caffeine Information, yet this information had not been supplied at the time of this measurement (i.e. at Pre-Beverage), therefore this numerical difference must be random. However, to ensure the significant interaction between Time and Caffeine Information in the main analysis was not an artefact of these numerical Pre-Beverage CWSQ differences, post-hoc 2 × 2 ANCOVAs with Priming and Caffeine Information as factors were run separately at Pre-Beverage and at Post-Beverage. At Pre-Beverage, there were no main effects of Priming (${F}_{(\mathrm{1,71})}$ = 0.004; *p* = 0.950, ${\eta }_{p}^{2}$ < 0.001) nor Caffeine Information (${F}_{(\mathrm{1,71})}$ = 2.987; *p* = 0.088, ${\eta }_{p}^{2}$ = 0.040), nor any interaction (${F}_{(\mathrm{1,71})}$ = 3.526; *p* = 0.065, ${\eta }_{p}^{2}$ = 0.047) on CWSQ scores. At Post-Beverage, there was no main effect of Priming on CWSQ score (${F}_{(\mathrm{1,71})}$ = 2.41; *p* = 0.125, ${\eta }_{p}^{2}$ = 0.033) but there was a significant main effect of Caffeine Information (${F}_{(\mathrm{1,71})}$ = 5.76; *p* = 0.019, ${\eta }_{p}^{2}$ = 0.075), whereby Post-Beverage CWSQ scores were significantly lower for participants Told Caffeine, and no interaction (${F}_{(\mathrm{1,71})}$ = 0.345; *p* = 0.559, ${\eta }_{p}^{2}$ = 0.005). Together, these results suggest that interaction between Time and Caffeine Information in the main analysis was not driven by pre-existing differences in CWSQ scores.

A pairwise comparison of Pre-Beverage CWSQ scores between the Gene + /Told Caffeine and Gene + /Told Decaf groups was performed, using the Scheffe procedure to control for type-1 error rate^39^. This test indicated a non-significant difference between the two groups (mean difference = 13.2, *p* = 0.093, 95% CI: −1.44–27.84).

There also appeared to be a difference in Pre-Post change in CWSQ scores between the Gene –/Told Decaf and the Gene +/Told Decaf group, however this change was also not significant when controlling for type-1 error using the Scheffe procedure (mean difference = 10.1, *p* = 0.209, 95% CI: −23.50–3.30).

### Effects of Belief in Caffeine Content of Beverage on CWSQ Difference Scores

In order to determine if strength of beliefs about whether the beverage was caffeinated or not predicted the degree of reduction of caffeine withdrawal symptoms, a ‘Post-Beverage – Pre-Beverage’ difference score was calculated, with a positive score indicating a reduction in withdrawal. In addition to the five covariates, this difference score was regressed on participants’ response to a question in the Exit Questionnaire indicating their strength of belief that the coffee they received was caffeinated or decaffeinated, with ‘7’ indicating that the coffee was ‘certainly caffeinated’ and ‘1’ indicating that is was ‘certainly decaffeinated’. When the five other covariates were controlled for the strength of belief that the beverage was caffeinated significantly predicted the magnitude of reduction in CWSQ scores (*t* = 5.209; *b* = 3.77; *SEb* = 0.723; *p* < 0.001) such that every 1-point increase in likelihood that beverage was caffeinated predicted a 3.77-point decrease in reported withdrawal symptoms.

### Secondary Outcomes: Blood Pressure and Concentration

No significant main-effects or interactions were observed for the two secondary outcome variables blood pressure and RVIP score.

## Discussion

These results provide support for the theory that beliefs concerning the presence or absence of a drug in the body can influence the way people perceive their withdrawal symptoms. Participants given decaffeinated coffee who were allowed to believe they had consumed caffeinated coffee showed a significantly greater reduction in reported caffeine withdrawal symptoms than participants who knew they had been given decaffeinated coffee. In addition degree of belief that they had consumed caffeine predicted level of self-reported withdrawal symptoms. These results confirm our previous findings^29^ and demonstrate that caffeine withdrawal, like withdrawal from other forms of addictive agents, is a syndrome whose interoceptive indices are sensitive to the influence of cognition.

The most interesting result, however, was that genetic information had no effect on caffeine withdrawal symptoms. Participants who were told over the phone that they had tested positive for the DRD2 allele reported levels of caffeine withdrawal after 24 hours of caffeine abstinence that were almost identical to those who were told they did not have the gene. This indicates that, at least in the domain of caffeine withdrawal, genetic risk information may not be sufficient to prime a nocebo effect in the same way as warnings of side-effects have been able to prime other nocebo effects such as insomnia^39^, gastrointestinal complaints^20^ and cognitive deficits in chemotherapy patients^17^.

The widespread accessibility of genetic testing is relatively new, as is research into the impacts of this testing. Only two prior studies have shown that being told one has tested positive for a gene related to a negative medical condition can induce nocebo-like effects. Dar-Nimrod and Colleagues^8^ found that ‘gene positive’ information can increase subjective distress, however it is unclear whether the distress was disease-specific or a non-volitional nocebo effect. Lineweaver and Colleagues^14^ found that being made aware of testing positive for a gene related to Alzheimer’s caused a significant decrease in performance on a memory test and in subjective ratings of memory compared to not being made aware. This is much closer to a true nocebo effect than Dar-Nimrod and Colleagues’^8^ finding, as the effect was observed in a disease-relevant domain. Why Lineweaver and Colleagues^14^ observed a nocebo priming effect when we did not is unknown. It is possible that, for the mostly elderly sample in Lineweaver and Colleagues’^14^ study, testing positive for a gene related to Alzheimer’s was a greater cause for concern, and perhaps thus a more potent prime, than a gene related to drug addiction and withdrawal was to our more age-heterogenous sample. Whatever the source of the difference in results, in light of our findings, the ability of genetic information to prime a condition-relevant response across all psychophysiological domains is questionable.

Importantly the Bayes Factor of 4.78 in favour of the null hypothesis, rather than simply the failure to find an effect, constitutes substantial evidence that genetic information does not affect caffeine withdrawal. Some results in the placebo field using priming manipulations have yielded remarkable results, such as lowering body-mass index and blood pressure^40^ and producing differences in ghrelin secretion^41, 42^. Not surprisingly, such findings have drawn considerable attention. However, the current results suggest that, rather than being ubiquitous, priming effects may be restricted to specific contexts and conditions. To this end, it is important to encourage researchers to publish the results of any adequately-designed studies on priming manipulations, irrespective of their outcomes, so that we can determine the characteristics of the prime and the conditions for which priming has an effect.

From visual inspection of Fig. 2. it appeared as if there may have been a difference in Pre- to Post-Beverage reduction in caffeine withdrawal between the Gene –/Told Decaf and the Gene +/Told Decaf groups. However, it is hard to imagine a genuine genetic priming effect that is specific to *recovery* from withdrawal, not actual withdrawal symptoms during abstinence, and only for participants who are told they have consumed decaffeinated coffee. A post-hoc pairwise comparison indicated no significant difference in the size of reduction in caffeine withdrawal between these two groups.

Several limitations to the study are worth mentioning. First, when a priming manipulation fails, it is always possible to question whether the prime was ‘strong enough’. Thanks to genetic essentialism, a bogus genetic test is likely a fairly significant experience to the individual, at least relative to simple verbal information. However, it is still possible that stronger manipulations might generate a priming effect. Second, participants’ expectancies of caffeine withdrawal before and after the receipt of the genetic information were not measured. Logically a positive genetic result should not have led to conscious expectancies of a *change* in severity of withdrawal symptoms because, implicitly, knowledge of having the gene means knowing one has *always* had the gene. However it would be interesting to verify whether genetic information does actually influence conscious expectancy, particularly in the current case where no effect on the target outcome was observed. Future studies in this area should include pre-post measures of conscious expectancies to determine whether these expectancies change and have any influence on condition-relevant symptoms. Thirdly it should be noted that the decaffeinated coffee used in the study does contain trace amounts of caffeine (4 mg or less per 180-ml cup, compared to 85–100 mg per 180-ml cup of ground caffeinated coffee). While there is a possibility that this small dose of caffeine reduced the ability of the prime to differentially affect *change* in caffeine withdrawal following the consumption of the coffee, in participants whose daily caffeine intake was ≥270 mg per day such a small dose of caffeine is unlikely to have resulted in any notable pharmacological buffering against primed withdrawal-reversal effects. Lastly, the lack of a measure of the level of concern participants had about the condition linked to the gene being tested may limit the generalisability of these results to other genetic tests for more serious conditions. Future studies should also include such a measure.

In conclusion this study found no effect of genetic priming on caffeine withdrawal symptoms, which was confirmed by a Bayesian analysis. As such the current study suggests that the effects of genetic priming are not ubiquitous and may instead be limited to specific conditions. Importantly the absence of an effect of the genetic prime in our study occurred despite the fact that caffeine withdrawal was shown to be sensitive to the effects of expectancy. That is, participants told they received caffeine at test had fewer withdrawal symptoms than those told they received no caffeine, despite both receiving decaffeinated coffee. Future research should seek to characterize the specific conditions that are sensitive to genetic priming effects.
